# Tumor Area Positivity (TAP) score of programmed death-ligand 1 (PD-L1): a novel visual estimation method for combined tumor cell and immune cell scoring

**DOI:** 10.1186/s13000-023-01318-8

**Published:** 2023-04-19

**Authors:** Chunyan Liu, Fang Fang, Ying Kong, Ehab A. ElGabry

**Affiliations:** Roche Tissue Diagnostics, 1910 E. Innovation Park Dr., Oro Valley, Tucson, AZ 85755 USA

**Keywords:** Programmed death-ligand 1 (PD-L1), Tumor area positivity, Tumor cells, Immune cells, Scoring method

## Abstract

**Background:**

Determination of programmed death-ligand 1 (PD-L1) protein expression level in tumor cells and tumor-associated immune cells is critical for identifying patients eligible for immunotherapy. PD-L1 manual scoring algorithms can generally be divided into two categories: cell counting and visual estimation. Cell counting can be time-consuming and is not in sync with pathology practice, which classically uses a Gestalt approach based on pattern recognition and visual estimation. In this study, we introduce the Tumor Area Positivity (TAP) score, which is a novel, straightforward method for scoring tumor cells and immune cells together using visual estimation.

**Methods:**

To demonstrate the reproducibility of TAP scoring among pathologists, between- and within-reader precision studies were performed both within (internal) and outside of (external) our organization. We also compared the TAP score to the Combined Positive Score (CPS), which is based on cell counting, for concordance and time efficacy.

**Results:**

The average positive agreement, average negative agreement, and overall percent agreement between and within readers were all above 85% for both internal and combined external reader precision studies. TAP score had high concordance rate at 1% and 5% cutoff compared with CPS at cutoff 1: positive percent agreement, negative percent agreement, and overall percent agreement were all above 85%.

**Conclusions:**

Our study showed the TAP scoring method to be straightforward, significantly less time-consuming, and highly reproducible with a high concordance rate between TAP score and CPS.

**Supplementary Information:**

The online version contains supplementary material available at 10.1186/s13000-023-01318-8.

## Introduction

The discovery of immune checkpoints has led to a paradigm shift toward immunotherapy treatment in cancer. One such checkpoint is the programmed cell death protein 1 (PD-1)/programmed death-ligand 1 (PD-L1) axis which is responsible for inhibiting an immune response of immune cells (IC) to foreign antigens [1]. Tumor cells (TC) can also express PD-L1, leading to activation of the PD-1/PD-L1 pathway, which subsequently allows TC to evade the immune response and results in tumor growth [1–3]. Increased PD-L1 expression in tissue from patients with cancer is positively correlated with clinical response to immunotherapy [4, 5]; this highlights the need for scoring methods to accurately quantify PD-L1 protein expression. Optimal scoring methods should be accurate, precise, and help simplify workflow for practicing pathologists. Currently, United States Food and Drug Administration (FDA)-approved PD-L1 immunohistochemistry (IHC) assays/algorithms include scoring methods that consider TC positivity and/or IC positivity (Table 1) [6–14]. Combined Positive Score (CPS) is the only FDA-approved method that combines TC and IC; however, it is an approach based on cell counting which is time consuming and not intuitive to practicing pathologists. In this study, we introduce the Tumor Area Positivity (TAP) score, a simple, visual-based method for scoring TC and IC together which addresses the limitations of a cell-counting approach with comparable efficacy and reproducibility.

**Table 1:**
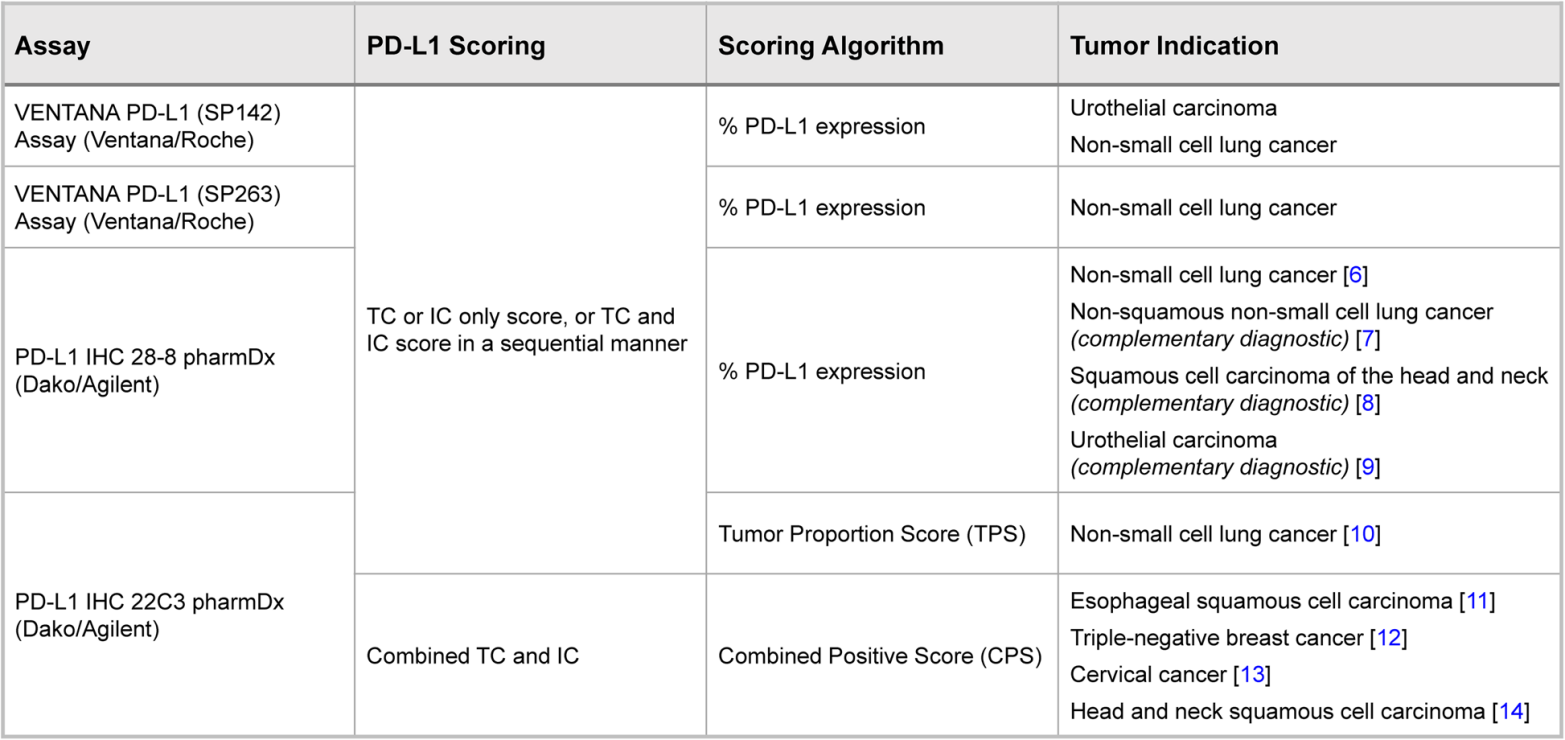
Summary of on-market FDA-approved PD-L1 IHC scoring algorithms and associated testing assays

This summary is for on-market PMA-approved PD-L1 IHC companion and complementary diagnostic tests and their associated scoring algorithms. Tests are companion diagnostics unless otherwise noted as complementary diagnostics

*FDA* United States Food and Drug Administration, *IC* Immune cells, *IHC* Immunohistochemistry, *PD-L1* Programmed death-ligand 1, *PMA* Premarket approval, *TC* Tumor cells

## Materials and methods

Institutional review board approval was obtained by the Roche Tissue Diagnostics Clinical Operation Department. The two reader precision studies used commercial samples. For the samples used in the comparison study, which were collected as part of a BeiGene study, consent was obtained in compliance with requirements. Each pathologist received training on the TAP scoring algorithm:

$$\text{TAP}=\frac{\%\text{PD-L1 positive TC and IC}}{\text{Tumor area}}$$

Pathologists were then required to pass a series of tests before participation in the studies (see Pathologist training section). Samples from gastric adenocarcinoma, gastroesophageal junction (GEJ) adenocarcinoma and esophageal squamous cell carcinoma (ESCC) (including both resections and biopsies) were stained using the VENTANA PD-L1 (SP263) assay (Ventana Medical Systems, Inc., Tucson, AZ, USA). Between- and within-reader precision studies were performed for the TAP score among three internal (Roche Tissue Diagnostics) pathologists (internal study) and six pathologists from three external organizations (external study). After successful completion of the reader precision studies, TAP score was compared to CPS retrospectively for concordance and time efficacy.

### TAP scoring method description and approach

#### Identification of tumor area

**Fig. 1:**
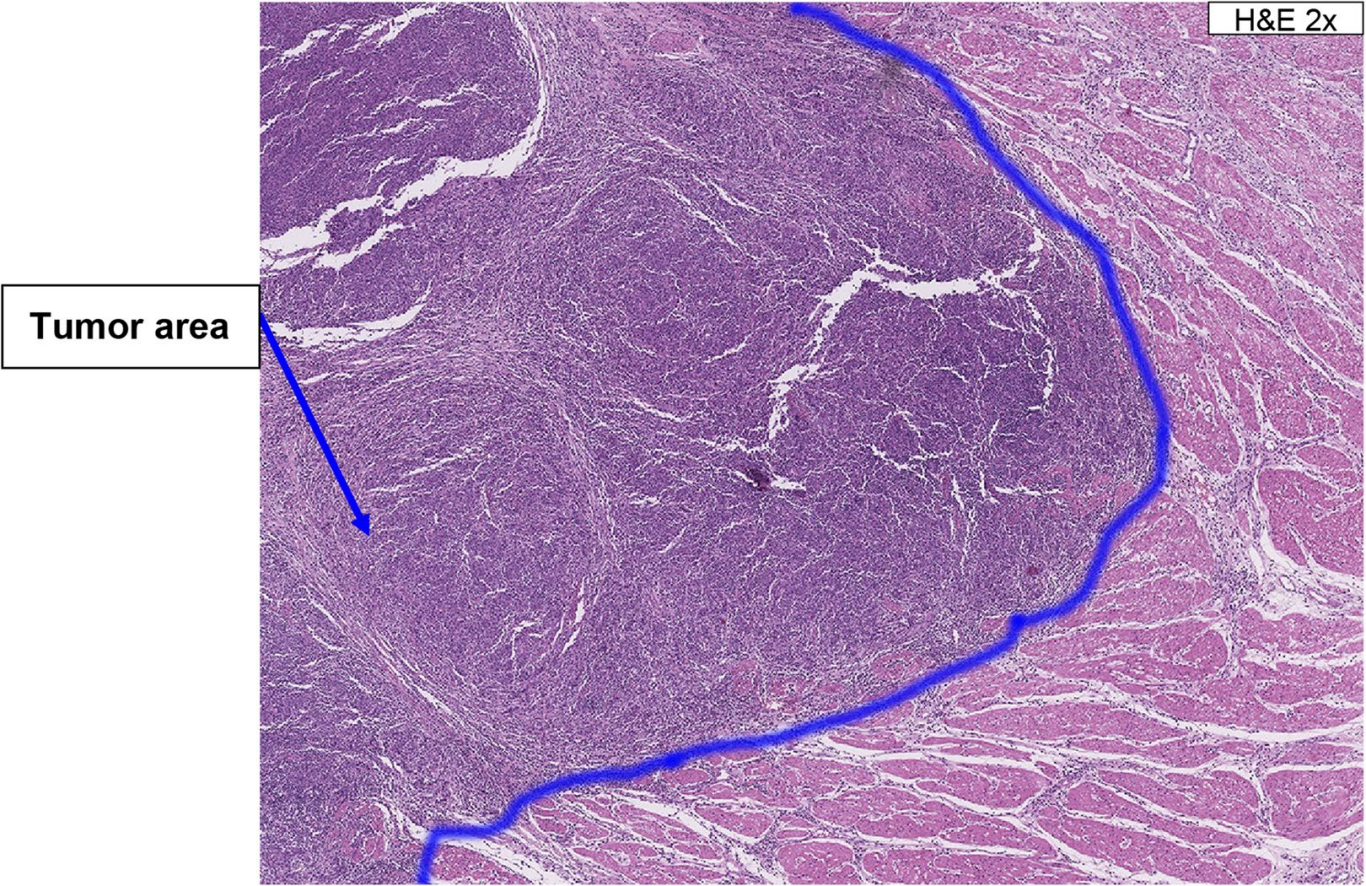
Identification of tumor area. Tumor area is defined as the area occupied by all viable TC and the tumor-associated stroma containing tumor-associated IC. *H&E* hematoxylin and eosin, *IC* immune cells, *TC* tumor cells

**Fig. 2:**
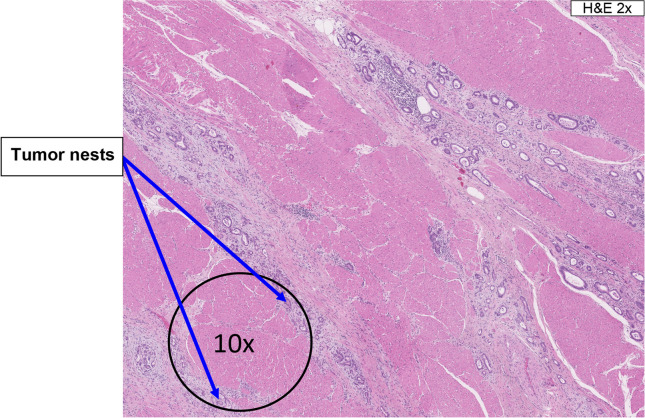
10x field rule: if tumor nests are separated by non-neoplastic tissue, they are included as part of the tumor area as long as the tumor nests are bordered on both sides of a 10x field; the intervening non-neoplastic tissue is also included in the tumor area. *H&E* hematoxylin and eosin

To determine TAP score, a hematoxylin and eosin-stained slide is first examined to identify tumor area (area occupied by all viable TC and the tumor-associated stroma containing tumor-associated IC) (Fig. 1). If tumor nests are separated by non-neoplastic tissue, they are included as part of the tumor area as long as the tumor nests are bordered on both sides of a 10x field; the intervening non-neoplastic tissue is also included in the tumor area (abbreviated as 10x field rule in the text below; Fig. 2). Necrosis, crush, and cautery artifacts are excluded from tumor area. For gastric and GEJ adenocarcinoma, the following must be considered:

- Pools of mucin and glandular luminal spaces in the presence or absence of viable TC are included as part of the tumor area.
- Tumor nests within the lymphovascular spaces are included in the tumor area.

##### *Tumor area determination in lymph nodes*

- For lymph nodes with multiple nests of tumor metastasis, apply the 10x field rule.
- In lymph nodes with focal or discrete tumor metastases, tumor area includes tumor nests and the areas occupied by the IC immediately adjacent to the leading edge of the metastatic tumor nests.

#### Determination of tumor-associated IC

Tumor-associated IC are intra- and peri-tumoral, including those present within the tumor proper, between tumor nests, and within any tumor-associated reactive stroma. In lymph nodes with focal or discrete tumor metastases, only IC immediately adjacent to the leading edge of the metastatic tumor nest were defined as tumor-associated IC.

#### Determination of TAP score

**Fig. 3:**
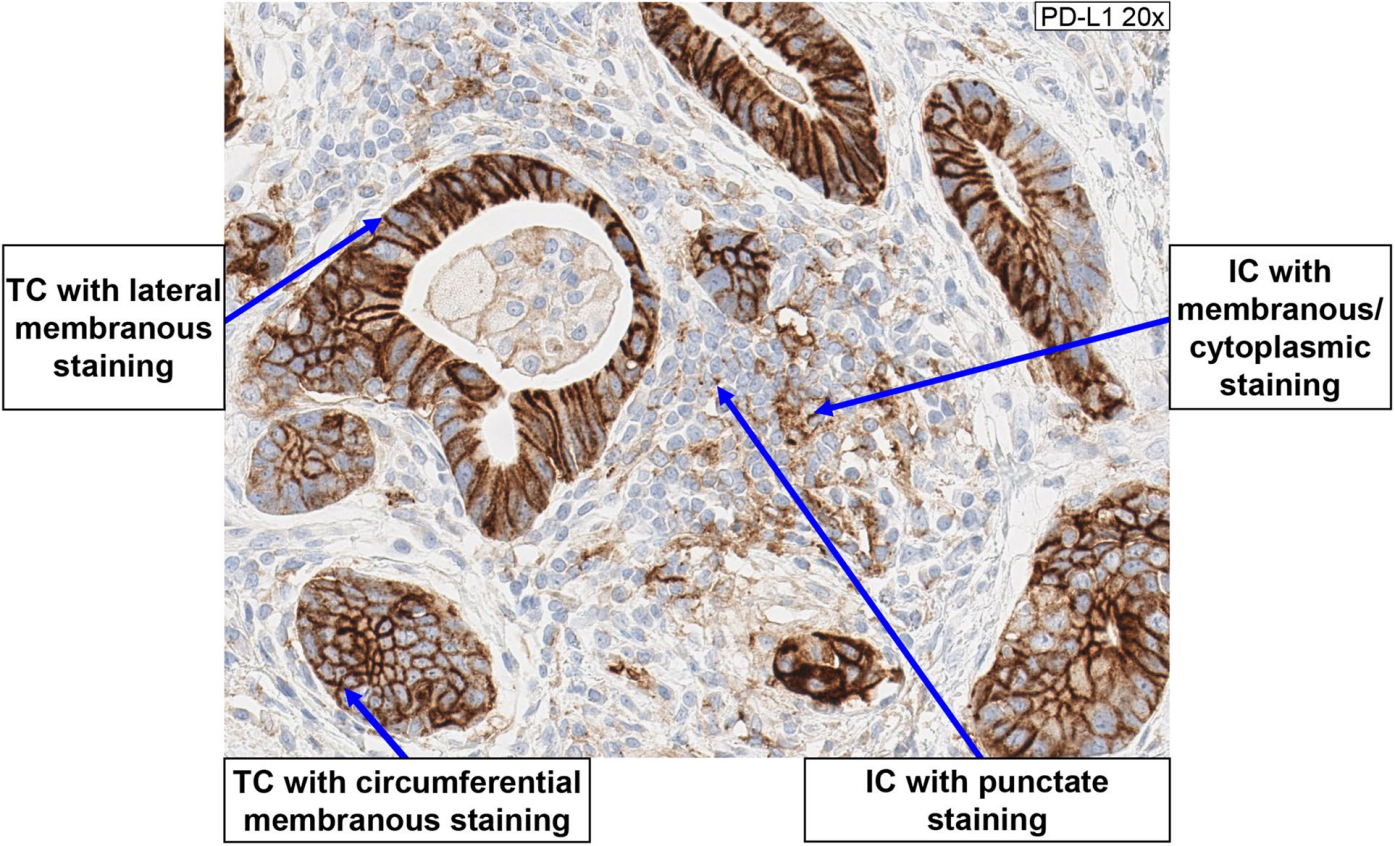
PD-L1 positive staining. Both circumferential and partial/lateral membrane staining of TC at any intensity is regarded as positive PD-L1 staining, while cytoplasmic staining of TC is disregarded; membranous, cytoplasmic, and punctate staining of tumor-associated IC at any intensity is regarded as PD-L1 positive staining. *IC* immune cells, *PD-L1* programmed death-ligand 1, *TC* tumor cells

The TAP score is determined on the IHC slide by visually aggregating/estimating the area covered by PD-L1 positive TC and tumor-associated IC relative to the total tumor area. Both circumferential and partial/lateral membrane staining of TC at any intensity is regarded as positive PD-L1 staining, while cytoplasmic staining of TC is disregarded; membranous, cytoplasmic, and punctate staining of tumor-associated IC at any intensity is regarded as PD-L1 positive staining [15] (Fig. 3).

For gastric and GEJ adenocarcinoma, staining of IC in the germinal center of lymphoid aggregates are included in the TAP score if they are located within the tumor area.

Intra-luminal macrophage staining is not included in the TAP score unless the macrophages completely fill the luminal space and are in direct contact with the TC. Staining of multi-nucleated giant cells, granulomas, and IC located within blood vessels and lymphatics are not included in the TAP score.

Off-target staining (e.g., fibroblasts, endothelial cells, neuroendocrine cells, smooth muscle, and nerves) should not be confused for specific PD-L1 staining, and is not included in the TAP score.

### Pathologist training

The training included review of an interpretation guide via Microsoft PowerPoint (Microsoft Corporation, Redmond, WA, USA) presentation, and review of a set of training glass slides using multi-headed microscopes in conjunction with the training pathologist. During the training session, PD-L1 biology, staining characteristics of TC and IC (Fig. 3), and acceptability of system level controls were reviewed, among other topics.

**Fig. 4:**
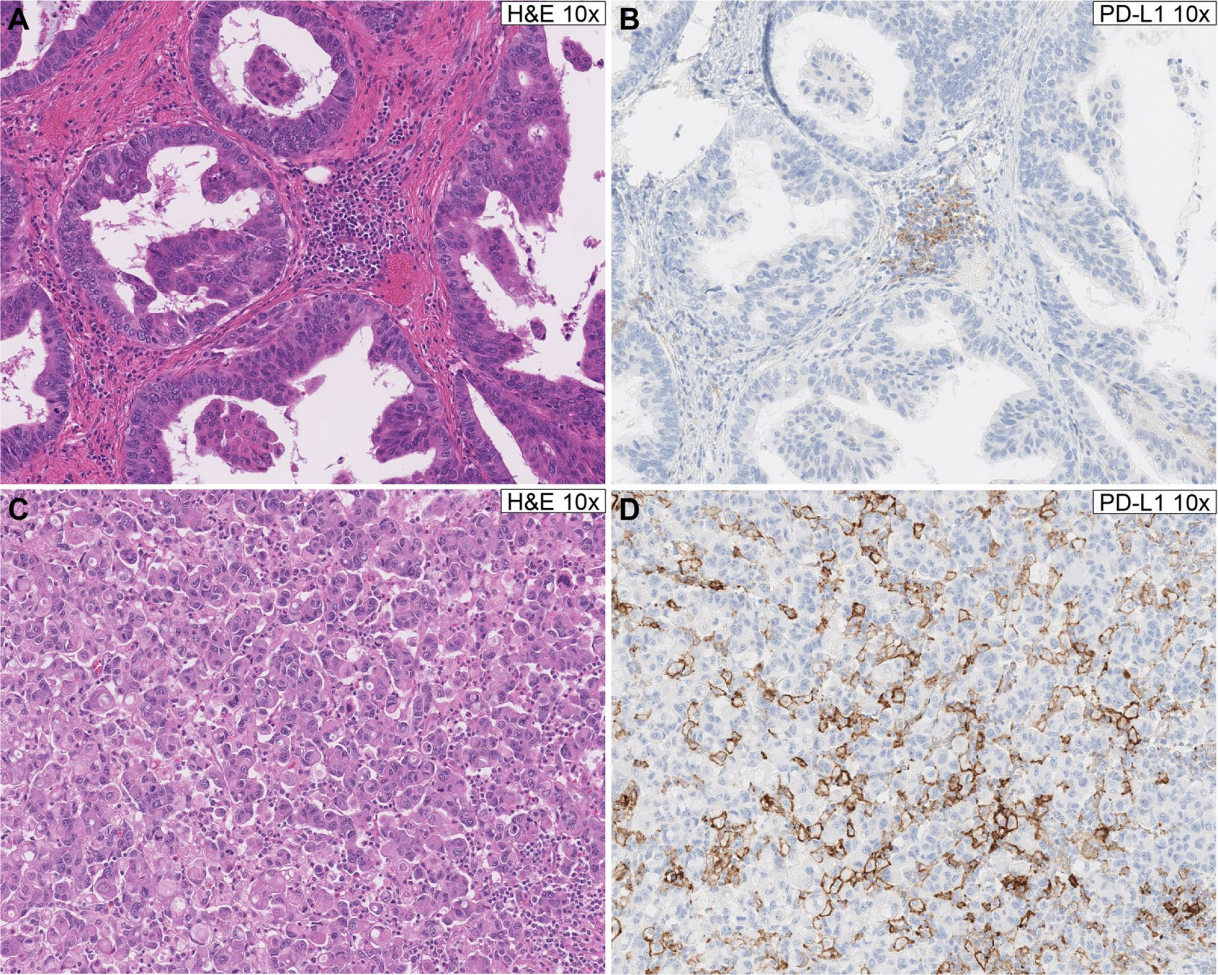
PD-L1 expression status around the 5% TAP score cutoff. **Top panel**: PD-L1 negative case, TAP score <5% (**A** H&E staining, **B** PD-L1 staining). **Bottom panel**: PD-L1 positive case, TAP score >5% (**C** H&E staining, **D** PD-L1 staining). *H&E* hematoxylin and eosin, *IC* immune cells, *PD-L1* programmed death-ligand 1, *TAP* Tumor Area Positivity

For gastric/GEJ adenocarcinoma, the test and training sets were designed to train the pathologists to accurately score PD-L1 expression status around the 5% cutoff (Fig. 4). The tests included a self-study set of 10 cases with consensus scores, a mini-test of 10 cases, and a final test of 60 cases. To pass the final test, the trainee pathologist had to achieve 85% agreement with reference scores on either an initial or a repeat test. The training on ESCC scoring was conducted using different training and test sets.

### Internal reader precision study

Three internal pathologists were trained and qualified for this study. This study evaluated: i) between-reader precision: across qualified readers individually evaluating the same set of randomized gastric or GEJ adenocarcinoma samples (*N* = 100 with equal distribution of PD-L1 expression level for positive [*n* = 50] and negative [*n* = 50] samples, spanning the range of the TAP score); and ii) within-reader precision: within individual readers evaluating the same set of gastric or GEJ adenocarcinoma samples over two assessments, separated by a wash-out time period of at least 2 weeks, and re-randomized and blinded prior to the second read. Between- and within-reader precision were assessed by evaluating the concordance of PD-L1 expression level of samples among the three readers from their first round of reads and within individual readers from their first and second round of reads, respectively. In the between-reader precision analysis, there were three pair-wise comparisons for each sample (reader 1 vs. reader 2, reader 1 vs. reader 3, and reader 2 vs. reader 3). With *N* = 100 samples, there were a total of 300 pair-wise comparisons. In the within-reader precision analysis, with *N* = 100 samples, there were 100 comparisons between the two reading rounds for each reader. All samples were commercially obtained formalin-fixed paraffin-embedded specimens.

A cutoff of 5%, using the TAP score, was used to determine if the PD-L1 expression in the sample was considered positive or negative. The sample set included 90% resection samples and 10% biopsy samples, 10% of which showed borderline range of PD-L1 expression. A sample was considered negative borderline if the TAP score was 2–4%, and positive borderline if TAP score was 5–9%. The average positive agreement (APA), average negative agreement (ANA), and overall percent agreement (OPA) between and within readers were then calculated, along with 95% confidence intervals (CIs). The acceptance criterion for between-reader precision was ≥85% ANA and APA. The acceptance criteria for within-reader precision were ≥ 90% OPA, and ≥ 85% ANA and APA. The assay was required to produce acceptable levels of non-specific staining on BenchMark ULTRA instruments (Ventana Medical Systems Inc.) in at least 90% of samples.

### External reader precision study

Three external organizations participated in an inter-laboratory reproducibility study using a cutoff of 5% TAP. At each site, two trained and qualified pathologists were selected to score the slides originating from the same sets of blocks. Specifically, 28 commercially obtained gastric or GEJ adenocarcinoma formalin-fixed paraffin-embedded specimens spanning the range of the TAP score were used in the external study. There was an equal distribution of PD-L1 expression level for positive (*n* = 14) and negative (*n* = 14) samples using the TAP score at 5% cutoff. Ten percent biopsy samples and 10% borderline cases were included in the sample set. The 28 cases were stained on five non-consecutive days over a period of at least 20 days at three sites, generating a total of five sets of slides for evaluation by the two pathologists at each site. The APA, ANA, and OPA were calculated across the three sites.

### Comparison of TAP and CPS

Gastric or GEJ adenocarcinoma and ESCC samples (*n* = 52) from a BGB-A317 trial carried out by BeiGene (Beijing, China) were used to compare the TAP and CPS scoring algorithms for evaluation of PD-L1 expression in a retrospective manner. Of the 52 samples, *n* = 10 were resection samples and *n* = 42 were biopsies. All samples were stained with the VENTANA PD-L1 (SP263) assay. The samples were distributed among eight internal pathologists and were scored using both methods. All eight pathologists were trained and qualified to evaluate PD-L1 expression using both the TAP and CPS scoring algorithms. The concordance of the TAP score at a 1% and 5% cutoff was assessed against a CPS score of 1 (equivalent to 1%), the FDA-approved cutoff for gastric or GEJ adenocarcinoma. The time spent on scoring for each method was also assessed.

## Results

### Internal reader precision study

As shown in Table 2, for between-reader analyses (including borderline cases), the pre-defined acceptance criteria were met for APA (296/298 [99.3%]; 95% CI, 98.0–100.0), ANA (300/302 [99.3%]; 95% CI, 98.0–100.0), and OPA (298/300 [99.3%]; 95% CI, 98.0–100.0). For within-reader analyses (including borderline cases), the pre-defined acceptance criteria were met for APA (296/299 [99.0%]; 95% CI, 98.0–100.0), ANA (298/301 [99.0%]; 95% CI, 98.0–100.0), and OPA (297/300 [99.0%]; 95% CI, 98.0–100.0). The background acceptability rate (600/600 [100.0%]; 95% CI, 99.4–100.0) also met the pre-defined acceptance criteria.

**Table 2:** TAP scoring precision for determining PD-L1 expression level in the internal study (including borderline cases)

Study	Statistics	Agreement	Results^**a**^
**Between-reader**	APA %	99.3	Pass
	*n/N*	296/298	
	95% CI	98.0–100.0	
	ANA %	99.3	Pass
	*n/N*	300/302	
	95% CI	98.0–100.0	
	OPA %	99.3	Pass
	*n/N*	298/300	
	95% CI	98.0–100.0	
**Within-reader**	APA %	99.0	Pass
	*n/N*	296/299	
	95% CI	98.0–100.0	
	ANA %	99.0	Pass
	*n/N*	298/301	
	95% CI	98.0–100.0	
	OPA %	99.0	Pass
	*n/N*	297/300	
	95% CI	98.0–100.0	
**Background**	OPA %	100.0	Pass
	*n/N*	600/600	
	95% CI	99.4–100.0	

Counts indicate the number of pairwise comparisons and do not represent the number of unique cases

*ANA* Average negative agreement, *APA* Average positive agreement, *CI* Confidence interval, *OPA* Overall percent agreement, *PD-L1* Programmed death-ligand 1, *TAP* Tumor Area Positivity

^a^The acceptance criteria for between-reader precision were ≥ 85% ANA and APA; the acceptance criteria for within-reader precision were ≥ 90% OPA, and ≥ 85% ANA and APA. The acceptance criterion for background staining was that the assay must produce acceptable levels of non-specific staining on ULTRA instruments in at least 90% of samples

### External reader precision study

Table 3 shows that site A achieved the lowest agreement rates for APA (88/109 [80.7%], 95% CI, 63.6–93.5), ANA (144/165 [87.3%], 95% CI, 78.0–95.7), and OPA (116/137 [84.7%], 95% CI, 73.2–94.9), while sites B and C produced identical results for APA (140/140 [100.0%], 95% CI, 97.3–100.0), ANA (140/140 [100.0%], 95% CI, 97.3–100.0), and OPA (140/140 [100.0%], 95% CI, 97.3–100.0). Overall, high agreement levels were demonstrated across the three sites (APA, 368/389 [94.6%], 95% CI, 90.8–98.0; ANA, 424/445 [95.3%], 95% CI, 91.5–98.5; OPA, 396/417 [95.0%], 95% CI, 91.2–98.3).

**Table 3:** TAP scoring precision for determining PD-L1 expression level in the external study (including borderline cases)

Strata	Statistics	Agreement
**Site A**	APA %	80.7
	*n/N*	88/109
	95% CI	63.6–93.5
	ANA %	87.3
	*n/N*	144/165
	95% CI	78.0–95.7
	OPA %	84.7
	*n/N*	116/137
	95% CI	73.2–94.9
**Site B**	APA %	100.0
	*n/N*	140/140
	95% CI	97.3–100.0
	ANA %	100.0
	*n/N*	140/140
	95% CI	97.3–100.0
	OPA %	100.0
	*n/N*	140/140
	95% CI	97.3–100.0
**Site C**	APA %	100.0
	*n/N*	140/140
	95% CI	97.3–100.0
	ANA %	100.0
	*n/N*	140/140
	95% CI	97.3–100.0
	OPA %	100.0
	*n/N*	140/140
	95% CI	97.3–100.0
**Combined**	APA %	94.6
	*n/N*	368/389
	95% CI	90.8–98.0
	ANA %	95.3
	*n/N*	424/445
	95% CI	91.5–98.5
	OPA %	95.0
	*n/N*	396/417
	95% CI	91.2–98.3

Counts indicate the number of pairwise comparisons and do not represent the number of unique cases

*ANA* Average negative agreement, *APA* Average positive agreement, *CI* Confidence interval, *OPA* Overall percent agreement, *PD-L1* Programmed death-ligand 1, *TAP* Tumor Area Positivity

### Correlation of TAP and CPS

The percentage agreement between TAP (1% cutoff) vs CPS (cutoff of 1) was 39/39 samples (100%; 95% CI, 91.0–100.0) for positive percent agreement (PPA), 11/13 samples (84.6%; 95% CI, 57.8–95.7) for negative percent agreement (NPA), and 50/52 samples (96.2%; 95% CI, 87.0–98.9) for OPA (Table 4). For TAP (5% cutoff) vs CPS (cutoff of 1), the percentage agreement was 35/39 samples (89.7%; 95% CI, 76.4–95.9) for PPA, 13/13 samples (100%; 95% CI, 77.2–100.0) for NPA, and 48/52 samples (92.3%; 95% CI, 81.8–97.0) for OPA (Table 5). The average time spent on scoring was 5 min for the TAP score and 30 min for the CPS scoring algorithm.

**Table 4:** Agreement between TAP (1% cutoff) and CPS (cutoff of 1) scoring algorithms

Statistics	Agreement
**PPA %**	100.0
***n/N***	39/39
**95% CI**	91.0–100.0
**NPA %**	84.6
***n/N***	11/13
**95% CI**	57.8–95.7
**OPA %**	96.2
***n/N***	50/52
**95% CI**	87.0–98.9

*CPS* Combined Positive Score, *NPA* Negative percent agreement, *OPA* Overall percent agreement, *PPA* Positive percent agreement, *TAP* Tumor Area Positivity

**Table 5:** Agreement between TAP (5% cutoff) and CPS (cutoff of 1) scoring algorithms

Statistics	Agreement
**PPA %**	89.7
***n/N***	35/39
**95% CI**	76.4–95.9
**NPA %**	100.0
***n/N***	13/13
**95% CI**	77.2–100.0
**OPA %**	92.3
***n/N***	48/52
**95% CI**	81.8–97.0

*CPS* Combined Positive Score, *NPA* Negative percent agreement, *OPA* Overall percent agreement, *PPA* Positive percent agreement, *TAP* Tumor Area Positivity

## Discussion

Understanding of immune checkpoint inhibitors has revolutionized the treatment options for cancer patients. Thus far, PD-L1 has been the focus of that recent paradigm shift. However, different scoring systems were introduced in a rapid successive fashion which may have burdened practicing pathologists who had to consistently play catch-up. This study aimed to provide a simple, visual-based estimate scoring method which combines TC and IC to identify the intended patient population of interest.

On-market FDA-approved PD-L1 scoring algorithms can be classified into TC- or IC-only score, TC and IC score in a sequential manner, or combined TC/IC score (Table 1). In general, TC-only scoring methods have been favorably adopted by the pathology community [16], whereas IC scoring or sequential TC/IC scoring have been perceived as challenging. CPS is the only FDA-approved method that combines TC and IC. It is a cell counting-based approach where the number of PD-L1-stained cells (TC, lymphocytes, and macrophages) is divided by the total number of viable TC, multiplied by 100 [17]. Cell counting can be time-consuming and is not in sync with pathology practice, which classically uses a Gestalt approach based on visual pattern recognition and estimation. Our study found that the average time spent on scoring was 5 min for the TAP score and 30 min for the CPS scoring algorithm, with one case of a large resection taking up to 1 h using CPS. Accordingly, pathologists must develop strategies to cope with CPS scoring during busy practice periods due to the time-consuming nature of the cell counting process. From communicating with practicing pathologists in the field, these strategies include piecemeal scoring approaches for large tumor resection specimens with heterogeneous staining pattern, eyeballing when applying 20x rules which provide estimated tumor cell numbers, and using a standard cellularity table for TC numbers.

An added complexity of CPS scoring is assessment of the type of IC to be included in the count, which requires the pathologist to select only mononuclear IC [17]. The TAP scoring method is inclusive of all types of IC; therefore, pathologists need not exhaust themselves under high magnification to confirm a cell type. Increasingly, research has shown that granulocytes are part of the adaptive tumor immune response [18, 19]; we have also observed weak to moderate PD-L1 expression in neutrophils around TC (Supplementary Fig. 1). This evidence led to inclusion of granulocytes in development of the TAP method. To overly simplify, the TAP method is essentially “the percentage of relevant brown (positive cells) over blue (entire tumor areas on IHC slide)”.

In this study, we compared the percentage agreement between TAP (1% and 5% cutoff) and CPS (cutoff of 1) in gastric/GEJ adenocarcinoma and ESCC samples using the VENTANA PD-L1 (SP263) assay, to investigate whether the two scoring methods were interchangeable, and if so, at what cutoff. The PPA, NPA, and OPA of the two comparisons were equal to or greater than 85%, with TAP score at 1% cutoff having better concordance with CPS 1 compared with TAP score at 5%. This suggests that the two algorithms, when used at different cutoffs, could potentially identify the same population of patients. In theory, samples in which the tumor stroma does not comprise large portions of tumor areas, such as mucosal biopsy specimens, have even greater potential for higher concordance of the two scoring methods (TAP and CPS). In fact, a study evaluated associations and potential correlations with clinical efficacy of the PD-L1 SP263 assay scored with the TAP algorithm (referred to as TIC [Tumor and Immune Cell]) at 5% cutoff and the PD-L1 22C3 assay scored with the CPS algorithm at 1% cutoff in gastroesophageal adenocarcinoma. Both the SP263 assay (TAP scoring) and 22C3 assay (CPS scoring) aided in the identification of patients with gastroesophageal adenocarcinoma likely to benefit from tislelizumab [20].

A potential limitation of TAP scoring is in defining the tumor areas in situations where the specimens have complicated histology with various non-neoplastic cells present in between tumor cells. However, this becomes less problematic as a pathologist reviews more cases and gains more experience.

The introduction of another PD-L1 scoring method (TAP) to an already confused market could be perceived as a limitation. However, as we have demonstrated, this method can help reduce confusion by providing a viable path for simplifying and standardizing pathology practice without compromising accuracy of patient selection.

## Conclusion

The data in this study show that the TAP scoring method is as effective as the CPS method in detecting patients with positive PD-L1 expression, but substantially less time-consuming. In addition to being highly reproducible among different pathologists, it can potentially standardize the existing scoring methods that evaluate both TC and IC.

## Supplementary Information

**Additional file 1: Supplementary Fig. 1.** Neutrophils with weak cytoplasmic staining.

## Data Availability

The data that support the findings of this study are available from Roche Tissue Diagnostics but restrictions apply to the availability of these data, which were used under license for the current study, and so are not publicly available. Data are however available from the authors upon reasonable request and with permission of Roche Tissue Diagnostics.

## Abbreviations

ANA: Average negative agreement
APA: Average positive agreement
CI: Confidence interval
CPS: Combined Positive Score
ESCC: Esophageal squamous cell carcinoma
FDA: United States Food and Drug Administration
GEJ: Gastroesophageal junction
IC: Immune cells
IHC: Immunohistochemistry
OPA: Overall percent agreement
PD-1: Programmed cell death protein 1
PD-L1: Programmed death-ligand 1
TAP: Tumor Area Positivity
TC: Tumor cells
