# Supplementary material for: Tumor Area Positivity (TAP) score of programmed death-ligand 1 (PD-L1): a novel visual estimation method for combined tumor cell and immune cell scoring
Source: Diagn Pathol. 2023 Apr 19;18:48. doi: 10.1186/s13000-023-01318-8 (PMC10114344; doi:10.1186/s13000-023-01318-8)
Supplement: Supplementary file 1 — Additional file 1: Supplementary Fig. 1. Neutrophils with weak cytoplasmic staining. [file 13000_2023_1318_MOESM1_ESM.pdf]

Supplementary Material

Tumor Area Positivity (TAP) score of programmed death-ligand 1 (PD-L1): a novel visual estimation method for combined tumor cell and immune cell scoring

Chunyan Liu, MD, PhD[1], Fang Fang, PhD[1], Ying Kong, MD, PhD[1], Ehab A. ElGabry, MD[1]

[1]Roche Tissue Diagnostics, Tucson, AZ, USA

Supplementary Fig. 1 Neutrophils with weak cytoplasmic staining.

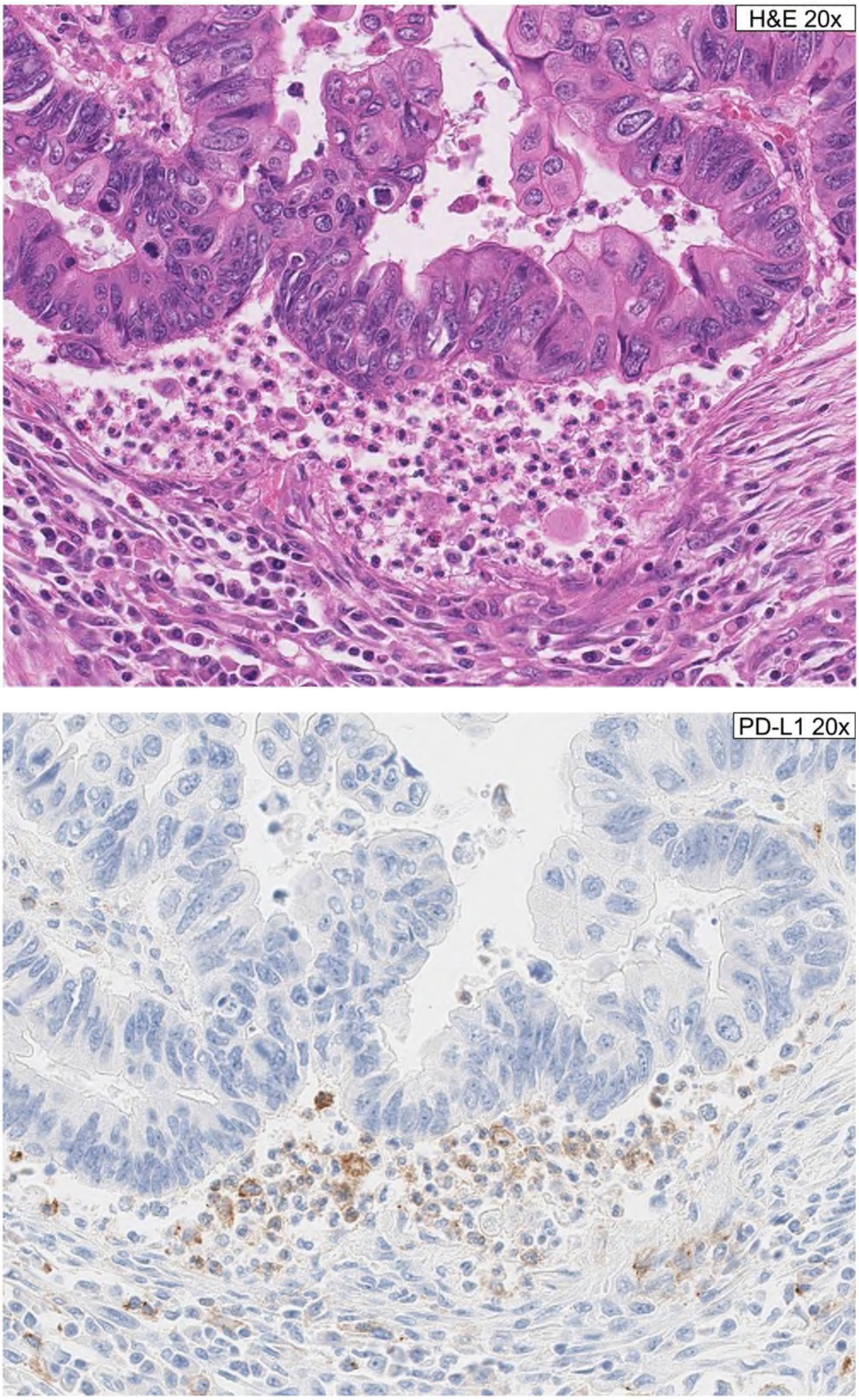

H&E hematoxylin and eosin, *PD-L1* programmed death-ligand 1
